# Throat Ail

**Published:** 1878-05

**Authors:** 


*Throat-Ail* gives a change of voice.
*Croup* gives difficult breathing.
*Bronchitis* gives a stuffed-up feeling.
*Consumption* gives steady emaciation.

Thus it is seen that throat-ail, or chronic laryngitis, is a disease at the top of the windpipe, where the voice organs are, its distinguishing feature being some change of the voice. Occasional additional feelings and symptoms are a huskiness of speech; sometimes the patient can only speak in the slightest whisper. Conversation is attended with an effort. Sometimes there is a painful feeling about the "swallow," a hurting sensation. At other times there is a pricking in the throat. Now and then these sensations extend up along the side of the neck towards the ear. An entire indisposition to talk is not unusual, for it requires an effort, or may excite cough. In almost all cases there is an everlasting disposition to heck and hem and clear the throat, present sometimes even in the sleep.

A gentleman applied to us in uncomplicated throat-ail; he could swallow no food; even liquids returned by the nose; the pain was terrible. He starved to death.

